# AIF-independent parthanatos in the pathogenesis of dry age-related macular degeneration

**DOI:** 10.1038/cddis.2016.437

**Published:** 2017-01-05

**Authors:** Ki-Hong Jang, Yun-Ju Do, Dongwon Son, Eunji Son, Jun-Sub Choi, Eunhee Kim

**Affiliations:** 1 Department of Biological Sciences, Chungnam National University, Yuseong-gu, Daejeon, Korea; 2 Catholic Institute for Visual Science, The Catholic University of Korea, #505 Banpo-dong, Seocho-gu, Seoul, Korea; 3 Graduate School of New Drug Discovery and Development, Chungnam National University, Yuseong-gu, Daejeon, Korea

## Abstract

Cell death of retinal pigment epithelium (RPE) is characterized as an essential late-stage phenomenon of dry age-related macular degeneration (AMD). The aim of this study was to elucidate the molecular mechanism underlying RPE cell death after exposure to oxidative stress, which occurs often because of the anatomical location of RPE cells. ARPE-19, an established RPE cell line, exhibited necrotic features involving poly (ADP-ribose) polymerase-1 (PARP-1) activation in response to hydrogen peroxide (H_2_O_2_). ARPE-19 cells were resistant to H_2_O_2_ when PARP-1 was depleted using siRNA or inhibited by a pharmacological inhibitor of PARP-1, olaparib. Our data suggest a causal relationship between PARP-1 activation and ARPE-19 cell death in response to H_2_O_2_. Next, we investigated downstream molecular events in PARP-1 activation. Increased mitochondrial depolarization, mitochondrial fission and alterations of the cellular energy dynamics with reduced NAD+ and ATP were observed in H_2_O_2_-treated ARPE-19 cells. H_2_O_2_-triggered mitochondrial dysfunction was inhibited by olaparib. Nevertheless, translocation of apoptosis-inducing factor (AIF), a biochemical signature for PARP-1-dependent cell death (parthanatos), was not observed in our study. Moreover, the depletion of AIF did not affect the amplitude of cell death, demonstrating the lack of a role for AIF in the death of ARPE-19 cells in response to H_2_O_2_. This feature distinguishes the type of death observed in this study from canonical parthanatos. Next, we examined the *in vivo* role of PARP-1 in a dry AMD animal model system. Histological analysis of the outer nuclear layer in the mouse retina revealed protection against sodium iodate (SI) following treatment with olaparib. Moreover, retina fundus and electroretinograms also confirmed such a protective effect in the SI-treated rabbit. Collectively, we report that AIF-independent PARP-1-dependent necrosis constitutes a major mechanism of RPE cell death leading to retinal degeneration in dry AMD.

Age-related macular degeneration (AMD) is the most common cause of blindness among the elderly.^1, 2^ AMD is classified into wet and dry forms; the dry form is more common than the wet form. Wet AMD is characterized by the generation of abnormal angiogenesis underneath the retina and leads to rapid vision loss. In contrast, retinal cells die progressively, displaying geographic atrophy (GA), in dry AMD. This gradual degeneration of retinal cells in GA patients also results in vision loss.^3, 4^ Fortunately, antiangiogenic therapeutics effectively delay the progression of wet AMD.^5, 6^ However, FDA-approved treatments for dry AMD are not available, although a few are now in clinical trials. Therefore, development of neuroprotective agents to maintain the remaining vision has been suggested as a future therapy for dry AMD.^7^

Retinal pigment epithelium (RPE), a monolayer of pigmented cells, is located between photoreceptor cells and Bruch's membrane and maintains retinal homeostasis via the transport of nutrients and waste, thereby protecting photoreceptor cells.^8^ The pathogenesis of dry AMD involves oxidative stress, mitochondrial dysfunction and inflammation.^9, 10, 11, 12, 13^ RPE cells are prone to exposure to high-energy light and rich polyunsaturated fatty acids, which are readily oxidized through photonic activation. Due to their anatomical localization and metabolic function, RPE cells are continuously exposed to chronic and cumulative oxidative stress and are most severely damaged in progressive dry AMD.^14^ RPE degeneration impairs retinal protective measures for the photoreceptor cells and results in their progressive death. To study the death mechanism of RPE cells, the human-derived RPE cell line, APRE-19, is often used as a cellular model upon oxidative stress^15, 16, 17, 18^ because these cells display properties that are commonly observed in RPE cells, such as morphological polarization and expression of the RPE-specific markers cellular retinaldehyde-binding protein and RPE65.^19^

The sodium iodate (SI) model is used to further understand the mechanism of RPE loss in dry AMD pathogenesis because SI is an oxidizing compound with specific toxicity for RPE and leads to alterations in RPE functions.^20, 21, 22, 23^ SI-induced retinal degeneration has been reported in various animal species, including sheep, rabbit and mice, with varying dose and administration routes.^20, 24, 25^ Moreover, SI damages the RPE through several mechanisms, including cross-reactivity with melanin, which converts glycine into toxic glucoxylate, inhibition of energy production enzymes and ROS accumulation.^26, 27, 28^ Therefore, we used SI-injected mice and rabbits to validate the *in vivo* role of PARP-1 in the pathogenesis of dry AMD.

Apoptosis and necrosis seem to be activated flexibly depending on the cell types and cellular context in the retina.^29^ When apoptosis is inhibited in photoreceptor cells, regulated necrotic death predominates, as if compensating for the absence of apoptosis. In this case, the sum of cell death remains relatively static despite the altered ratio of regulated necrosis to apoptosis. This compensation provides an explanation for therapeutic failure with single blockage of apoptosis to prevent retinal cell death. Therefore, necrotic death in the retinal cells has been studied extensively, and a combination therapy of apoptotic and necrotic inhibitors seems to be promising for the protection of retinal cells.

Poly (ADP-ribose) polymerases (PARPs) constitute a large family of enzymes that catalyze the transfer of ADP-ribose units onto target proteins. In humans, 17 members of the PARP family have been identified and share a conserved catalytic domain.^30^ PARP-1 is thought to have a critical role in cellular physiology because the majority (>90%) of PAR polymer synthesis derives from PARP-1.^30, 31, 32^ PARP-2 is the closest homolog to PARP-1, displaying 69% similarity in the catalytic domain, and PARP-1 and -2 are responsible for most of the PAR polymer synthesis.^33^ PARP-1 has multiple cellular roles, acting both in cell survival and in cell death pathways in cell type- and stimulus-dependent manners.^34, 35^

Activated PARP-1 under oxidative stress consumes NAD+ and depletes cellular ATP, eventually leading to cellular energy collapse.^36^ Moreover, PARP-1 activation results in the translocation of apoptosis-inducing factor (AIF) from mitochondria to the nucleus, fragmenting DNA.^37, 38^ Accumulation of such biochemical events completes PARP-1-mediated necrotic death of cells, parthanatos, which has been implicated in various age-related neurodegenerative diseases.^39, 40^ This observation led us to examine whether parthanatos is also involved in the pathogenesis of dry AMD, a prevalent senile eye disease.

Here, we demonstrate that PARP-1-mediated necrosis constitutes a substantial portion of the death of ARPE-19 cells in response to hydrogen peroxide (H_2_O_2_) insult. Mitochondrial dysfunction was observed downstream of PARP-1 activation. However, AIF was irrelevant in this death process, indicating that this type of cell death is distinct from canonical parthanatos in which AIF translocation follows PARP-1 activation. Moreover, we also validated the *in vivo* role of PARP-1 in retinal degeneration using Si-injected mice and rabbits. Collectively, this study reports the presence of an AIF-independent parthanatos pathway in the pathogenesis of dry AMD.

## Results

### H_2_O_2_ induces necrotic death in ARPE-19 cells

**Figure 1:**
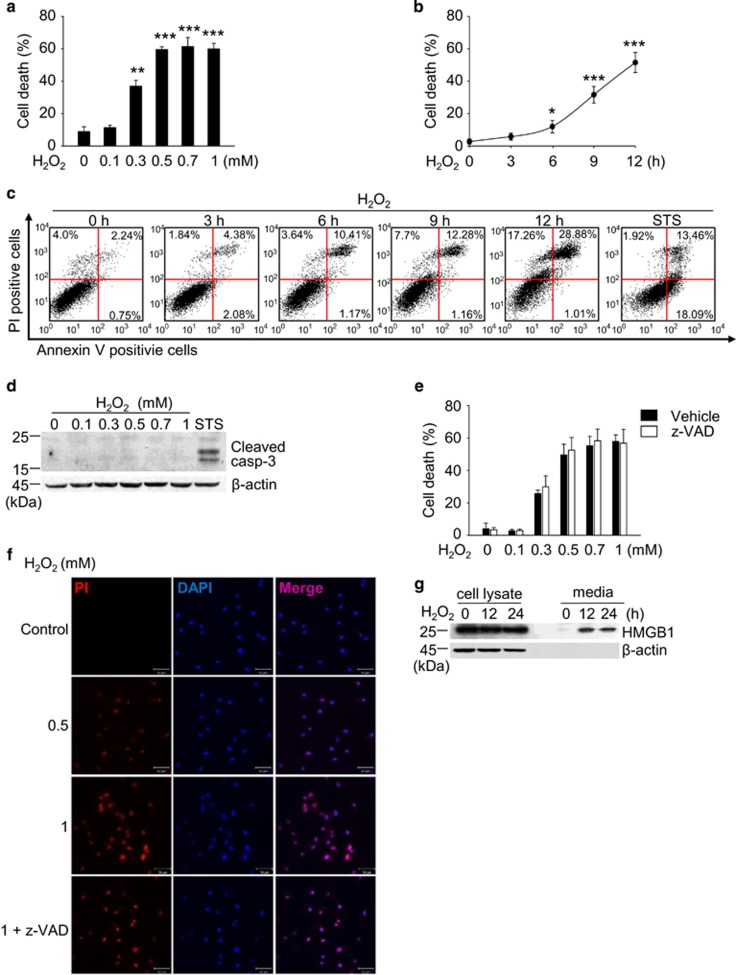
H_2_O_2_ induces necrotic death in ARPE-19 cells. (**a** and **b**) ARPE-19 cells were treated with indicated concentrations of H_2_O_2_ for 12 h (**a**) or 0.5 mM H_2_O_2_ for the indicated times (**b**). Cell death was analyzed by flow cytometry using propidium iodide (PI) staining. (**c**) Cells were treated with 0.5 mM H_2_O_2_ for the indicated times or 1 *μ*M STS for 12 h. Cells were also analyzed by flow cytometry using double staining with Annexin V and PI. Annexin V-negative/PI-positive (upper left) cells represent necrosis, double-positive cells (upper right) represent late apoptosis, and Annexin V-positive/PI-negative cells (lower right) represent the early stage of apoptosis. (**d**) Cells were treated with the indicated concentrations of H_2_O_2_ or 1 *μ*M STS for 8 h. Cleavage of caspase-3 was analyzed by western blotting. (**e**) Cells were treated with 0.5 mM H_2_O_2_ in the presence or absence of 50 *μ*M z-VAD for 12 h, and cell death was detected by flow cytometry. (**f**) Cells were seeded on poly-d-lysine-coated coverslips and treated with the indicated concentrations of H_2_O_2_ with or without 10 *μ*M z-VAD for 12 h. The nuclei were stained with PI and 4′,6-diamidino-2-phenylindole (DAPI). Representative confocal microscopic images are shown. (**g**) Cells were treated with 0.5 mM H_2_O_2_ for the indicated times. The release of high-mobility group box 1 (HMGB1) and cellular HMGB1 expression were detected by western blotting. The values are the mean±S.D. from triplicate independent experiments; *P<0.05, **P<0.01, ***P<0.001

ARPE-19 cells were treated with H_2_O_2_ at concentrations of 1 *μ*M up to 1 mM. Exposure to lower concentrations of H_2_O_2_ (1–100 *μ*M) did not induce cell death in ARPE-19 cells (Supplementary Figure 1). In contrast, ARPE-19 cells died in dose- and time-dependent manners at pathophysiological concentration ranges reported in diverse diseases^41, 42^ (Figures 1a and b). Because ARPE-19 cells are proximal to the choroid, we examined cell death following exposure to H_2_O_2_ in other cells distal to the choroid, retinal ganglion cells (RGC-5). RGC-5 cells were more sensitive to H_2_O_2_ compared with ARPE-19 cells (Supplementary Figure 2), suggesting that distance from the choroid might correlate with retinal cellular sensitivities to oxygen. Next, we performed flow cytometry to elucidate the predominant form of death in ARPE-19 cells following exposure to H_2_O_2_. Propidium iodide (PI) single-positive cells progressed to PI/Annexin V double-positive cells in a time-dependent manner (Figure 1c). Conversely, the population of Annexin V single-positive cells were not altered throughout the assessed time period. Moreover, caspase-3 was not activated by up to 1 mM H_2_O_2_ (Figure 1d). This finding implies that necrotic death occurred under the above conditions. In another set of experiment, Annexin V-positive cells increased with caspase-3 activation following the administration of staurosporine (STS), demonstrating that the apoptotic machinery in ARPE-19 cells was intact (Figures 1c and d). Furthermore, z-VAD treatment did not protect ARPE-19 cells from the H_2_O_2_ insult, indicating that apoptotic death did not occur (Figure 1e). Necrotic death in ARPE-19 cells was further confirmed using confocal microscopy (Figure 1f) and by the release of high-mobility group box 1, a marker of necrosis, into the medium (Figure 1g). Collectively, our results clearly demonstrate that necrotic death occurred in ARPE-19 cells in response to H_2_O_2_.

### PARP-1 activation mediates H_2_O_2_-induced necrotic death in ARPE-19 cells

We next investigated which type of regulated necrosis occurred in the H_2_O_2_-induced death of ARPE-19 cells. Based on the compensatory role of regulated necrosis when apoptosis is blocked, we examined the involvement of necroptosis, which is one type of regulated necrosis, in response to H_2_O_2_ treatment of ARPE-19 cells. The necroptosis inhibitor necrostatin-1^43, 44, 45^ and siRNA-mediated genetic depletion of receptor-interacting protein kinase 1 (RIPK1) did not protect ARPE-19 cells from the H_2_O_2_ insult, implying that necroptosis was not involved in H_2_O_2_-induced ARPE-19 cell death (Supplementary Figures 3a–d). Furthermore, RIPK3, an indicator of responsiveness to necroptosis,^46, 47^ was not expressed in ARPE-19 cells (Supplementary Figure 3e).

**Figure 2:**
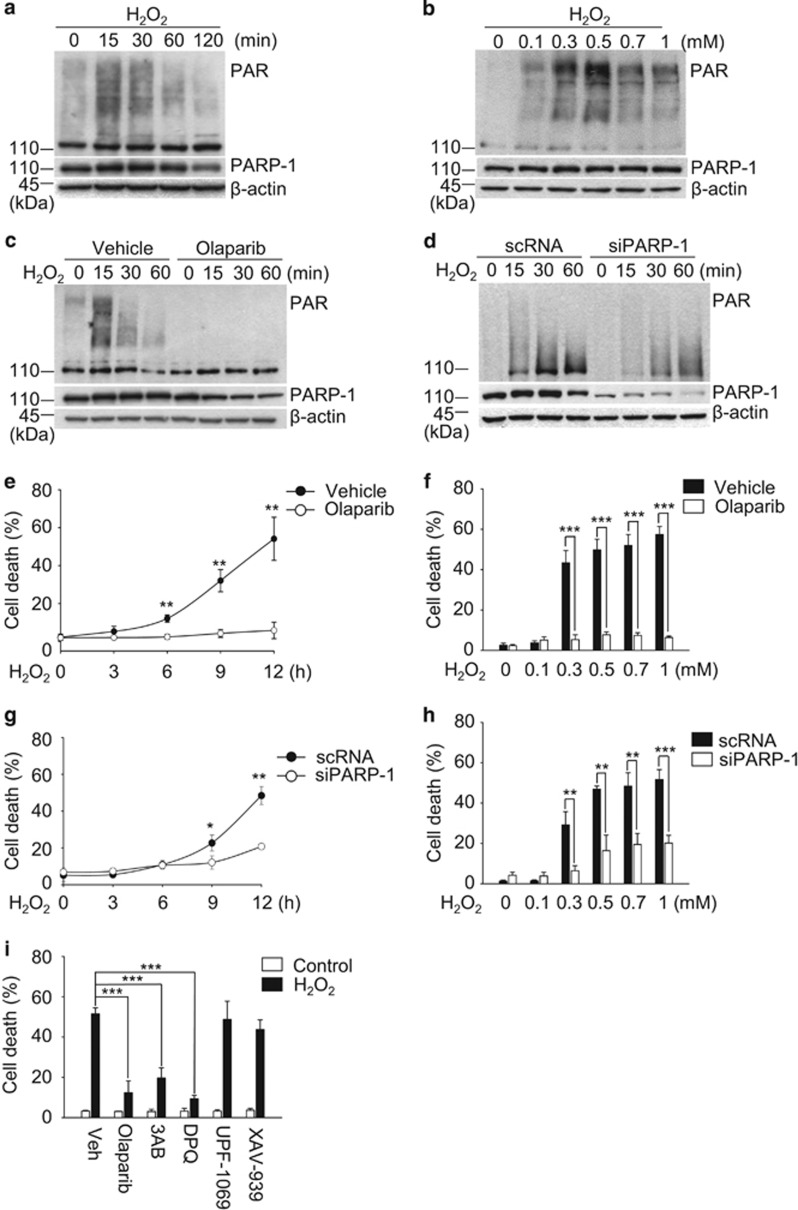
PARP-1 activation mediates H_2_O_2_-induced necrotic death in ARPE-19 cells. (**a** and **b**) ARPE-19 cells were treated with 0.5 mM H_2_O_2_ for the indicated times (**a**) or the indicated concentrations of H_2_O_2_ for 15 min (**b**). Western blot analysis revealed the expression of PARP-1 and PAR. (**c**) Cells were treated with 0.5 mM H_2_O_2_ for the indicated times with or without 10 *μ*M olaparib. The expression levels of PARP-1 and PAR were detected by western blotting. (**d**) ARPE-19 cells were transfected with scrambled-siRNA (scRNA) or PARP-1 targeting siRNA (siPARP-1 #3) for 48 h. Next, the cells were treated with 0.5 mM H_2_O_2_ for the indicated times. Immunoblotting revealed the cellular expression levels of PARP-1 and PAR. (**e** and **f**) Cells were treated with 0.5 mM H_2_O_2_ for the indicated times (**e**) or the indicated concentrations of H_2_O_2_ for 12 h (**f**) in the presence or absence of 10 *μ*M olaparib. Cell death was measured by flow cytometry. (**g** and **h**) Cells were transfected with scRNA or siPARP-1 for 48 h and subsequently treated with 0.5 mM H_2_O_2_ for the indicated times (**g**) or the indicated concentrations of H_2_O_2_ for 12 h (**h**). Cell death was evaluated by flow cytometry. (**i**) Cells were treated with 0.5 mM H_2_O_2_ for 12 h in the presence or absence of PARP inhibitors: 1 *μ*M olaparib, 50 *μ*M 3AB, 30 *μ*M DPQ, 1 *μ*M UPF-1069 and 1 *μ*M XAV-939. The values are the mean±S.D. from triplicate independent experiments; *P<0.05, **P<0.01, ***P<0.001

Thereafter, we examined whether parthanatos, another type of regulated necrosis, participates in H_2_O_2_-induced death of APRE-19 cells. We found that H_2_O_2_ treatment induced the synthesis of cellular poly ADP-ribose (PAR) polymers (Figures 2a and b). The PAR polymer synthesis was completely blocked by the pharmacological inhibitor of PARP-1, olaparib (Figure 2c). PAR polymer synthesis also decreased following the genetic knockdown of PARP-1 using the siRNA system (Supplementary Figure 11a; Figure 2d). Moreover, H_2_O_2_ increased death in time- and dose-dependent manners, and olaparib inhibited H_2_O_2_-induced death in ARPE-19 cells (Figures 2e and f). Similarly, the genetic depletion of PARP-1 protected ARPE-19 cells from the H_2_O_2_ insult (Figures 2g and h). Furthermore, we analyzed the subtype of PARPs that was activated by H_2_O_2_ in ARPE-19 cells using various PARP inhibitors because PAR polymers can be synthesized by various PARP family members. The reagents 3AB and DPQ, inhibitors of PARP-1 and -2, prevented H_2_O_2_-induced ARPE-19 cell death, but UPF-1069 (a selective PARP-2 inhibitor) and XAV-939 (a selective PARP-5 inhibitor) did not (Figure 2i). Collectively, these data indicate that ARPE-19 cell death in response to H_2_O_2_ is mediated mainly by PARP-1. Next, we examined whether PARP-1-mediated death occurs in ARPE-19 cells in response to other oxidative stresses. In ARPE-19 cells, olaparib blocked 1-methyl-3-nitro-1-nitrosoguanidine (MNNG)-induced death but not *tert*-Butyl hydroperoxide (*t*-BHP)- and rotenone-induced death (Supplementary Figure 4a). These results indicate that MNNG triggers PARP-1-mediated death, but *t*-BHP and rotenone do not. PARP-1 activation by MNNG was further confirmed using western blot analysis of PAR (Supplementary Figure 4b). Taken together, our data show that PARP-1 is not a common mediator of APRE-19 cell death in response to diverse oxidative stresses.

### AIF is dispensable for H_2_O_2_-induced necrotic death through PARP-1 activation in ARPE-19 cells

**Figure 3:**
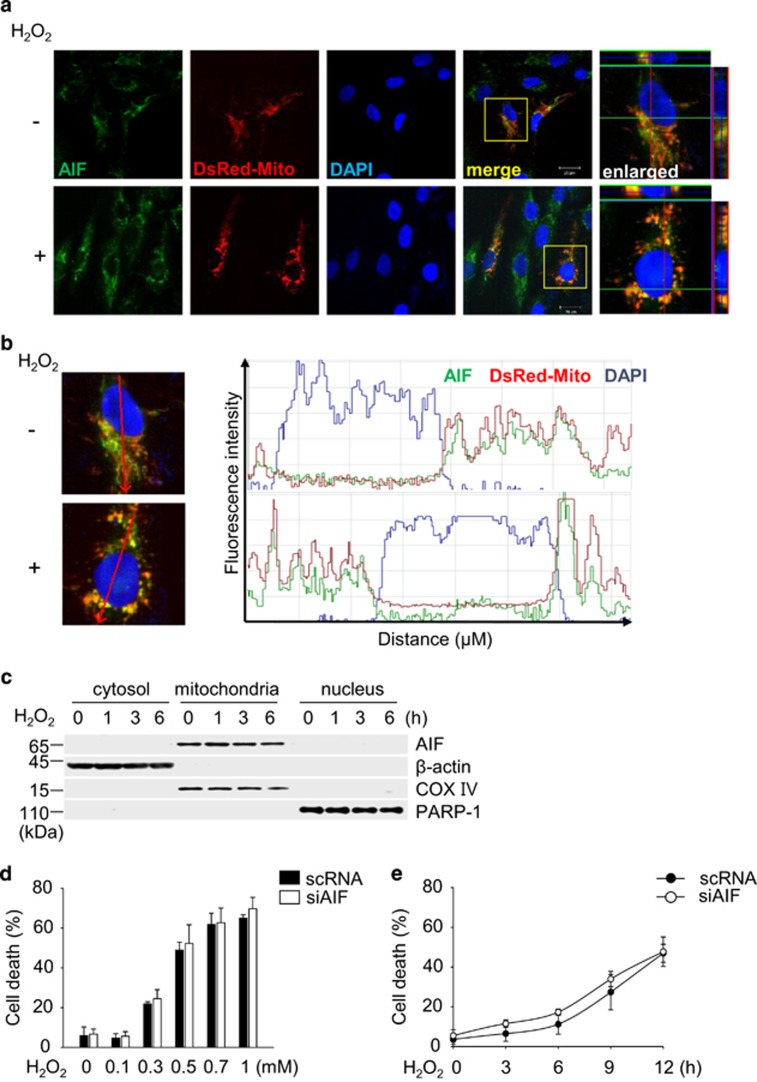
AIF is dispensable for H_2_O_2_-induced necrotic death through PARP-1 activation in ARPE-19 cells. (**a**) ARPE-19 cells were transfected with DsRed-Mito for 48 h to visualize mitochondria (red). Subsequently, the cells were treated with 0.5 mM H_2_O_2_ for 6 h and immunostained with anti-AIF antibody to determine the localization of AIF. The AIF signal is shown in green. The nuclei were counterstained with DAPI (blue). Representative fluorescence images (including z-stacks) are shown. (**b**) The graphs (right panel) show the fluorescence intensity profiles in three fluorescence channels along the arrow (left panel). (**c**) Cells were treated with 0.5 mM H_2_O_2_ for the indicated times. The cytosolic, mitochondrial, and nuclear fractions were recovered and examined using an immunoblot assay with anti-AIF antibody. COX IV, *β*-actin and PARP-1 were used as mitochondria, cytosolic, and nuclear markers, respectively. (**d** and **e**) Cells were transfected with scRNA or AIF targeting siRNA (siAIF #1) for 48 h and then exposed to H_2_O_2_ at the indicated concentrations for 12 h (**d**) or to 0.5 mM H_2_O_2_ for the indicated times (**e**). Cell death was measured by flow cytometry. The values are expressed as the mean±S.D. of three independent experiments

We next examined the translocation of AIF into the nucleus, a biochemical signature of parthanatos, in H_2_O_2_-insulted ARPE-19 cells. AIF remained in the mitochondria upon H_2_O_2_ insult by fluorescence intensity profile analysis (Figures 3a and b). Subcellular fractionation analysis using differential ultracentrifugation to determine the relative distribution of AIF also revealed no changes in the localization of AIF upon H_2_O_2_ insult (Figure 3c). Moreover, we also did not observe the genomic DNA fragmentation in H_2_O_2_-exposed ARPE-19 cells, further confirming lack of AIF translocation to the nucleus (Supplementary Figure 5). Nevertheless, mitochondrial fission was observed, revealing a punctate morphology in ARPE-19 cells (Figure 3a). Next, we investigated whether AIF translocates into the nucleus in another type of retinal cells, RGC-5, because ARPE-19 cells and RGC-5 cells showed differential sensitivities to H_2_O_2_ (Supplementary Figure 2). We also examined AIF translocation in another cell type, SH-SY5Y and mouse embryonic fibroblast (MEF), upon H_2_O_2_. AIF was released from the mitochondria and translocated into the nucleus in RGC-5, SH-SY5Y and MEF cells following the H_2_O_2_ insult and olaparib blocked AIF translocation (Supplementary Figure 6). Our results showed that different types of cells display distinctive downstream pathways following PARP-1 activation in response to H_2_O_2_. We further questioned whether the presence of AIF is dispensable to complete H_2_O_2_-induced ARPE-19 cell death. The H_2_O_2_ insult induced death in AIF-depleted ARPE-19 cells using the siRNA system (Supplementary Figure 11b; Figures 3d and e). This finding indicates that AIF is not required for H_2_O_2_-induced ARPE-19 cell death. In contrast, H_2_O_2_-induced death was attenuated by AIF depletion using siRNA system in RGC-5, SH-SY5Y and MEF cells (Supplementary Figure 7). Our findings indicate the presence of a novel necrotic pathway that is distinctive from canonical parthanatos in H_2_O_2_-exposed ARPE-19 cells.

### PARP-1 activation triggers mitochondrial dysfunction and cellular energy collapse in response to H_2_O_2_ insult

**Figure 4:**
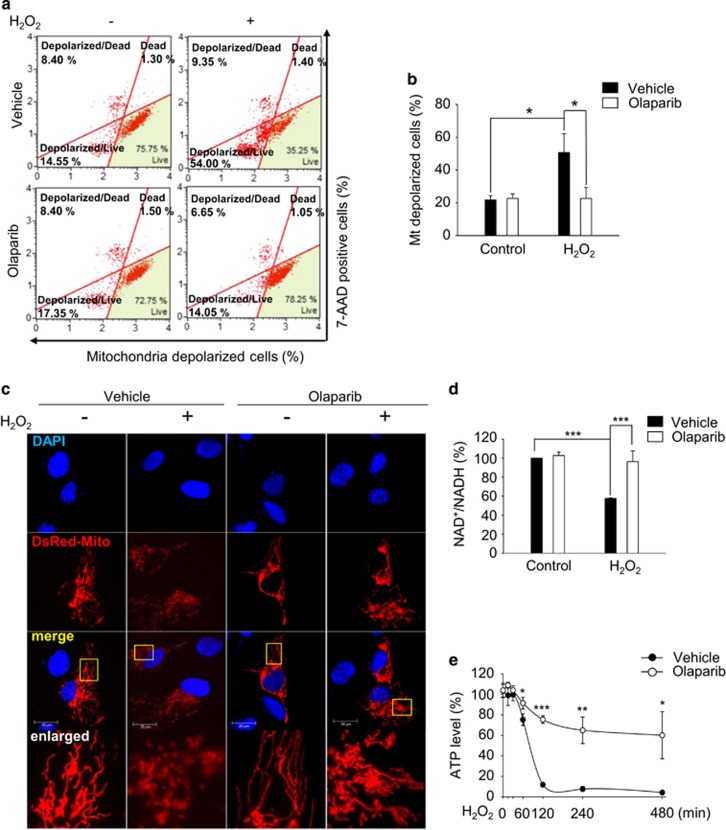
PARP-1 activation triggers mitochondrial dysfunction and cellular energy collapse in response to H_2_O_2_. (**a**) ARPE-19 cells were treated with 0.5 mM H_2_O_2_ with or without 10 *μ*M olaparib for 3 h, and the mitochondrial potential was measured using a Muse analyzer. The cells in the left quadrant are depolarized cells. (**b**) The graph was obtained from a quantitative analysis of depolarized cells. (**c**) Cells were transfected with DsRed-Mito for 48 h. Subsequently, the cells were treated with 0.5 mM H_2_O_2_ in the presence or absence of 10 *μ*M olaparib for 1 h. Fluorescence images show the alteration in mitochondrial morphology. (**d**) Cells were treated with 0.5 mM H_2_O_2_ in the presence or absence of 10 *μ*M olaparib for 4 h. The cellular level of NAD+ was determined using a VICTOR plate reader. (**e**) The cells were treated with 0.5 mM H_2_O_2_ with or without 10 *μ*M olaparib for the indicated times. The ATP level was detected using a VICTOR plate reader. The values are expressed as the mean±S.D. of three independent experiments. *P<0.05, **P<0.01, ***P<0.001

We examined whether PARP-1 activation damages mitochondria in H_2_O_2_-insulted ARPE-19 cells because mitochondrial defects are observed in the RPE of AMD eyes.^48^ H_2_O_2_ treatment induced mitochondrial depolarization in ARPE-19 cells when examined with MUSE analyzer (Figures 4a and b) and addition of NAD+ restored mitochondrial polarization (Supplementary Figure 8). Fluorescence images revealed an altered mitochondrial morphology and increased mitochondrial fission in ARPE-19 cells in response to H_2_O_2_ (Figure 4c). Moreover, long optic atrophy 1 isoform (OPA1^L^), a marker for mitochondrial fusion, gradually decreased in response to H_2_O_2_ (Supplementary Figure 9). The pharmacological inhibition of PARP-1 decreased cell depolarization (Figures 4a and b) and preserved mitochondrial morphology (Figure 4c) in ARPE-19 cells following exposure to H_2_O_2_. Therefore, our data clearly demonstrate that H_2_O_2_ damages mitochondria through PARP-1 activation in ARPE-19 cells. Next, we measured the cellular NAD+ and ATP levels following exposure to H_2_O_2_. The levels of NAD+ and ATP significantly decreased in response to H_2_O_2_, and this decrease was prevented by the pharmacological inhibition of PARP-1 in ARPE-19 cells (Figures 4d and e). These results indicate that activated PARP-1 by H_2_O_2_ triggers cellular energy depletion. Collectively, our findings suggest that PARP-1 activation upon an H_2_O_2_ insult provokes mitochondrial dysfunction and leads to energy failure with declines in cellular NAD+ and ATP in ARPE-19 cells.

### PARP-1 participates in retinal degeneration in a dry AMD mouse model

**Figure 5:**
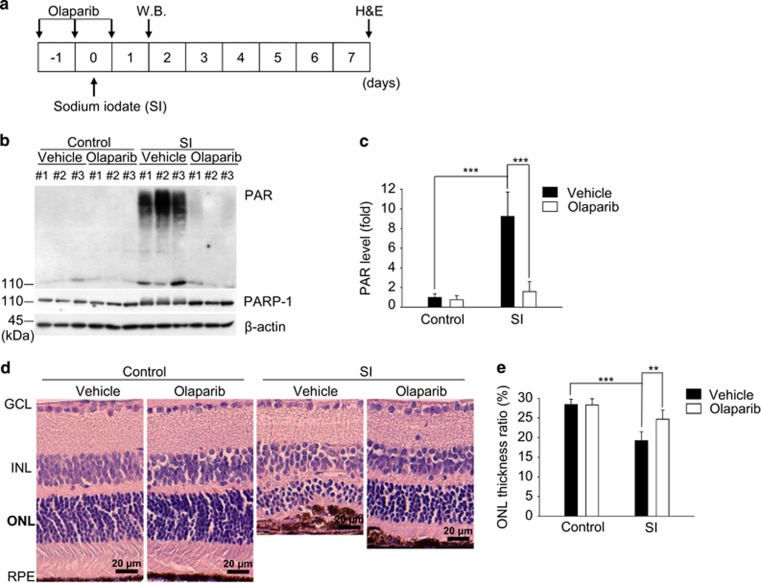
PARP-1 participates in retinal degeneration in a dry AMD mouse model. (**a**) Schematic diagram of the experimental design for the AMD model mouse via SI injection. Olaparib or saline administered three times with 24-h intervals. The mice were sacrificed 1 day and 7 days after the last injection of olaparib, respectively. (**b**) Immunoblot assay showing the cellular levels of PARP-1 and PAR in retinal lysates of mice in each experimental group. Each lane represents an individual mouse. (**c**) Quantitative analysis of the PAR polymer levels in the retina (*n*=6 eyes/group). (**d**) The enucleated eyes were stained with hematoxylin and eosin (H&E). Histological images showing the alterations in retinal morphology. (**e**) The graph shows the ratio of the outer nuclear layer (ONL) thickness to the total retinal thickness in H&E-stained samples (*n*=6 eyes/group). The thickness was measured using ImageJ software. The values are presented as the mean±S.D of three independent experiments. **P<0.01, ***P<0.001

To validate the role of PARP-1 in the pathogenesis of AMD *in vivo*, SI-injected mice were used as an animal model.^27, 49^ The administrative procedure is schematized in Figure 5a. The synthesis of PAR polymers was triggered in mice upon SI injection, reaching a maximum at day 1 (Supplementary Figures 10a and b), and this synthesis was blocked by olaparib (Figures 5b and c). Next, we performed a histological analysis using hematoxylin and eosin staining to investigate the effect of PARP-1 on retinal morphology in SI-injected mice. Alterations in retinal morphology began to appear 3 days after SI injection in mice with a reduction of the outer nuclear layer (Supplementary Figures 10c and d). In contrast, olaparib moderated the alterations in retinal morphology by preserving the outer nuclear layer thickness (Figures 5d and e). Collectively, our data show that PARP-1 mediates retinal degeneration in a dry AMD mouse model.

### PARP-1 inhibition preserves the physiological function of the retina in rabbits following SI insult

**Figure 6:**
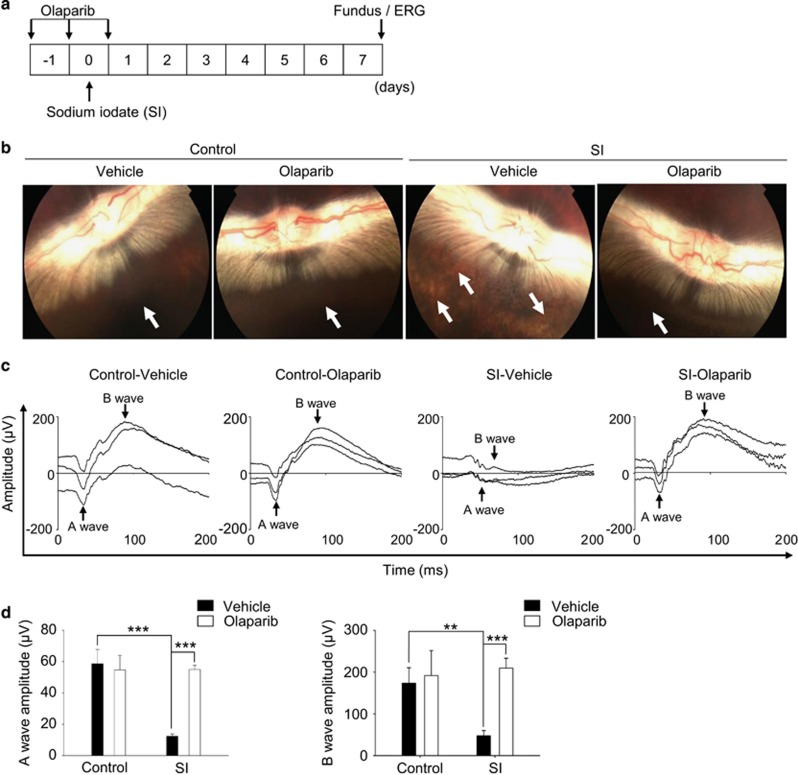
PARP-1 inhibition preserves the physiological function of the retina in the rabbit against SI insult. (**a**) Schematic diagram of the experimental design for the AMD rabbit model established by SI injection. Olaparib or saline administered three times with 24-h intervals. Fundoscopic examination and ERG were performed 7 days after the last injection of olaparib. (**b**) Representative fundus images showing the retinal condition of the rabbits in each experimental groups (*n*=6 eyes/group). (**c**) The ERG was used to investigate the function of the retina in response to light (*n*=3 eyes/group). Waveforms of the scotopic ERG response were provoked by a flash at 3000 mcd.s/m^2^. (**d**) The graphs were obtained by quantitative analysis of A- and B-wave amplitudes. The values are the mean±S.D. of three independent experiments. **P<0.01, ***P<0.001

To validate the *in vivo* role of PARP-1 in the physiological function of the retina, we performed a fundoscopic examination to assess the geographic atrophy in live animals. Fundoscopy showing the optic nerve regions was used in SI-injected rabbits because the large size of rabbit eyes facilitates this procedure. The administrative procedure is schematized in Figure 6a, and a representative fundus image is shown in Figure 6b. The retina fundus of SI-injected rabbits appeared to be brighter than that of the control rabbits, representing RPE loss. The brightness of the retina fundus was moderated by olaparib, implying that PARP-1 mediates the RPE loss in SI-injected rabbits (Figure 6b). Next, we evaluated the function of the retina using electroretinography in SI-injected rabbits to confirm the effect of PARP-1 in the pathogenesis of AMD. The amplitude of the A-wave was drastically reduced, demonstrating functional damage of the photoreceptors of SI-injected rabbits (Figures 6c and d).^50, 51^ Similarly, the amplitude of the B-wave also decreased, revealing dysfunction in the bipolar cells of SI-injected rabbits.^52, 53^ Olaparib conserved the amplitude of A- and B- waves similar to the control. The parameters of the electroretinogram (ERG) potential are summarized in Table 1. Collectively, these results show that PARP-1 impairs visual function in the pathogenesis of dry AMD.

## Discussion

This study shows that the PARP-1-mediated necrotic pathway is distinct from that of the canonical parthanatos, serving as a novel mechanism of RPE loss in the pathogenesis of dry AMD: (1) PARP-1 is activated, leading to the death of ARPE-19 cells through mitochondrial dysfunction and cellular energy collapse in response to H_2_O_2_; (2) AIF, a typical downstream effector of PARP-1, does not participate in the execution of cell death in H_2_O_2_-induced ARPE-19 cells; (3) the essentiality of PARP-1 was validated in mouse and rabbit dry AMD models.

The mechanism underlying oxidative stress-induced RPE loss in AMD pathogenesis remains unclear. Consistent with a previous study reporting low expression levels of the DNA fragmentation factor 45/40,^16^ we did not observe features of apoptosis in H_2_O_2_-treated ARPE-19 cells. Endogenous caspase-8 expression was also decreased in ARPE-19 cells compared with that in other ocular cells.^16^ Such a downregulation of apoptotic components might serve as a survival strategy to compensate for the rapid turnover of RPE cells.^54^ An attenuated cellular apoptotic potential would shift the cellular death pathway toward necrosis, in which substrates of caspases, including RIPK1 and PARP-1, have key roles, providing one explanation for the predominance of necrosis in ARPE-19 cells in response to oxidative stress.

It is noteworthy that PARP-1-mediated necrosis is activated, whereas RIPK1-mediated necroptosis is quiescent between the two regulated necrotic pathways. Inactivation of the necroptotic machinery in H_2_O_2_-exposed ARPE-19 cells seems to result from the lack of RIPK3 expression. Failure of necrosome formation due to the RIPK3 deficiency would preclude the completion of necroptosis, irrespective of RIPK1 expression. Such a restriction would be advantageous for longevity in post-mitotic RPE cells with limited regeneration potential.^55, 56, 57^ In contrast, PARP-1 participates in the cellular repair process and in necrosis. Therefore, retention of PARP-1 activity would serve dual measures for repair and cell death.

Our study has shown that genetic depletion of PARP-1 removed most PAR polymers in H_2_O_2_-treated ARPE-19 cells. Moreover, the pharmacological inhibition of PARP-1 sufficiently protected ARPE-19 cells against H_2_O_2_. Thus, PARP-1 has a major role in PAR polymer synthesis under oxidative stress in ARPE-19 cells. PARP-1 is highly conserved, especially in the contiguous 50-amino-acid sequence, the signature motif of PARP, in the catalytic domain, which displays 100% conservation in vertebrates and the most abundantly expressed isoforms among the PARP family members, supporting the importance of PARP-1.^30, 31, 32, 58, 59^ Therefore, our data support the use of PARP-1 as a target for the treatment of dry AMD.

RPEs located proximal to the choroidal vessels are frequently exposed to oxygen and thereby might have adapted to cope with oxidative stresses. The lack of AIF translocation into the nucleus upon exposure to H_2_O_2_ in ARPE-19 cells would interrupt one death pathway: apoptosis. Therefore, the blockage of AIF translocation would protect against nuclear damage. In contrast, RGCs distal to the choroidal vessel would not have to be rigorous to resolve alterations caused by oxidative damage. This observation seems to provide a physiological explanation for the translocation of AIF in RGC-5 cells in response to oxidative damage. Therefore, we speculate that differential sensitivities to H_2_O_2_ among retinal cells might derive from their anatomical positions.

Nuclear translocation of AIF following PARP-1 activation is the signature of parthanatos, and AIF is required as an executioner of parthanatos.^37, 60, 61, 62, 63^ The lack of AIF translocation with PARP-1 activation shown in our study has also been found in human renal proximal tubule epithelial cells.^64^ Furthermore, the lack of involvement of AIF in executing PARP-1-mediated death has been reported in MEF cells following exposure to MNNG.^65^ Considering the dual role of AIF, that is, mitochondrial protection and DNA fragmentation in the nucleus, AIF seems to function flexibly, depending on the context such as the cell type or the type of stimulus.

Several genes are involved in the pathogenesis of dry AMD. Moreover, clinical analysis of dry AMD patients has revealed a dysregulation of complement-associated genes.^66^ Therefore, it will be important to investigate the clinical relevance of PARP-1 in patients with dry AMD. Abundant PARP-1 inhibitors that have already been developed as therapeutics for other diseases would serve as repurposed drug candidates for the treatment of dry AMD.

## Materials and Methods

### Reagents

The following reagents were obtained commercially: rabbit anti-caspase-3 from Cell Signaling Technology (Danvers, MA, USA); rabbit anti-HMGB1 from Abcam (Cambridge, UK); mouse anti-*β*-actin, H_2_O_2_, *t*-BHP, propidium iodide (PI), STS, Necrostatin-1 (Nec-1), 3*-*Aminobenzamide (3AB), 3,4-dihydro-5-[4-(*1*-piperidinyl)butoxyl]-*1*(2H)-isoquinolinone (DPQ), poly-l-lysine, nicotinamide adenine dinucleotide (NAD), SI and 50% glutaraldehyde from Sigma-Aldrich (Saint Louis, MO, USA); horseradish peroxidase (HRP)-conjugated anti-mouse antibody, HRP-conjugated anti-rabbit antibody, fluorescein (FITC)-conjugated anti-mouse antibody and 4′,6-diamidino-2-phenylindole (DAPI) from Thermo Fisher Scientific (Rockford, IL, USA); mouse anti-RIPK1 antibody, mouse anti-PARP-1 antibody, and Annexin V from BD Biosciences (San Jose, CA, USA); rabbit anti-RIPK3 antibody and mouse anti-AIF antibody from Santa Cruz Biotechnology (Dallas, TX, USA); rabbit anti-PAR antibody from Trevigen (Gaithersburg, MD, USA); mouse anti-PAR antibody from Enzo Life Sciences (Farmingdale, NY, USA); z-VAD-fmk from Millipore (Darmstadt, Germany); olaparib from Selleck Chemicals (Houston, TX, USA); UPF-1069 and XAV-939 from Tocris Bioscience (Minneapolis, MN, USA); and MNNG from Tokyo Chemical Industry (Tokyo, Japan).

### Cell culture

Human cultured RPE (ARPE-19) cells and retinal ganglion cells (RGC-5) were cultured in a humidified atmosphere (37 °C, 5% CO_2_) in Dulbecco's Modified Eagle's Medium (DMEM, WelGENE, Daegu, Korea)/F12 supplemented with 10% fetal bovine serum (Atlas Biologicals, Fort Collins, CO, USA) and 1% penicillin/streptomycin (Thermo Fisher Scientific). Human neuroblastoma cell line (SH-SY5Y) and MEF were cultured in DMEM supplemented with 10% fetal bovine serum at the same condition as ARPE-19 cells.

### Flow cytometry

ARPE-19 cells were treated with the indicated concentrations of H_2_O_2_ for 12 h or 0.5 mM H_2_O_2_ for the indicated times. The cells were harvested and stained with PI at a final concentration of 5 *μ*g/ml. Cell death was analyzed using a Guava easyCyte flow cytometer (Millipore). In another set of experiments, H_2_O_2_-treated ARPE-19 cells were harvested and washed using Annexin V buffer provided by the supplier (BD Biosciences) and then stained with Annexin V. Next, PI was added at a final concentration of 5 *μ*g/ml. The cells were then evaluated using a Guava easyCyte flow cytometer and quantified using InCyte software (Millipore).

### Fluorescence and immunofluorescence

ARPE-19 cells were cultured on poly-l-lysine-coated coverslips in 12-well plates. The cells were treated with 0.5 mM H_2_O_2_ in the presence or absence of 10 *μ*M z-VAD for 1 h. Subsequently, the cells were stained with 5 *μ*g/ml PI for 5 min to detect necrotic death, and the nuclei were stained with DAPI for 5 min. To observe the mitochondrial morphology, ARPE-19 cells were transiently transfected with DsRed-Mito (Clontech Laboratories, Mountain View, CA, USA). After 48 h of transfection, the ARPE-19 cells were treated with 0.5 mM H_2_O_2_ with or without 10 *μ*M olaparib. The cells were fixed with 4% paraformaldehyde for 15 min and subsequently permeabilized for 10 min using 0.01% Triton X-100. The nuclei were stained with DAPI for 5 min. For immunofluorescence, ARPE-19 cells were treated with 0.5 mM H_2_O_2_ for 1 h. The cells were fixed with 4% paraformaldehyde for 15 min and permeabilized with 0.01% Triton X-100 for 10 min. The cells were incubated with anti-AIF antibody overnight at 4 °C and then washed three times with cold PBS for 10 min each. Solutions with fluorescent dye-conjugated secondary antibody were added to the cells for 2 h at room temperature, and the nuclei were stained with DAPI. The coverslips of all samples were mounted onto microscope slides using fluorescence-mounting medium (Dako, Carpinteria, CA, USA). All samples were analyzed using a Zeiss LSM 510 laser scanning confocal microscope (Carl Zeiss, Oberkochen, Germany).

### Western blot analysis

ARPE-19 cells were lysed in lysis buffer (20 mM Tris–HCl pH 7.5 containing 150 mM NaCl, 2 mM EDTA, 1% NP-40, 0.4 mM PMSF, 25 mM *β*-glycerophosphate, 1 mM Na_3_VO_4_, 1 mM DTT and 1 mM NaF) and then centrifuged at 12 000 × *g* for 10 min at 4 °C. The protein concentrations were determined by the Bradford method using the Bio-Rad protein assay kit (Bio-Rad, Hercules, CA, USA). The samples were separated by SDS-PAGE and transferred to a nitrocellulose membrane. The membranes were blocked with 5% skim milk and incubated with suitable primary antibodies at 4 °C for 12 h. After three washes for 10 min each, the membranes were incubated with HRP-conjugated secondary anti-mouse or anti-rabbit antibody. The protein bands were visualized using an ECL detection kit (YounginFrontier, An-Yang, Korea).

### HMGB1 release analysis

The conditioned media from H_2_O_2_-treated ARPE-19 cells were used to detect secreted HMGB1 in the media. The conditioned media were filtered and concentrated using Amicon ultra-4 devices (Millipore) according to the manufacturer's protocol. All samples were analyzed by western blotting.

### Subcellular fractionation

APRE-19 cells were harvested and washed with TD buffer (2.5 mM Tris–HCl pH 7.6 containing 13.5 mM NaCl, 0.5 mM KCl) and subsequently centrifuged at 700 × *g* for 5 min at 4 °C. The pellets were resuspended in CaRSB buffer (10 mM Tris–HCl pH 7.5 containing 10 mM NaCl, 1.5 mM CaCl_2_, 2 mM DTT, protease inhibitor) and homogenized using 26-Gauge syringes. The homogenized samples were added to 2.5 × Mannitol-Sucrose buffer (MS buffer, Tris–HCl pH 7.6 containing 210 mM Mannitol, 70 mM Sucrose, 5 mM EDTA, 2 mM DTT, protease inhibitor) and centrifuged at 700 × g for 7 min at 4 °C. The supernatants contained cytosolic components, including mitochondria, and the pellets contained nuclear components. First, the supernatants were added to 1–1.5 M discontinuous sucrose gradient solutions (10 mM HEPES pH 8.0, 5 mM EDTA, 1 M and 1.5 M sucrose) and then centrifuged at 38 000 × *g* for 30 min at 4 °C. The aspirated band between 1 and 1.5 M sucrose was washed with 1 × MS buffer and then lysed in lysis buffer (mitochondria). Second, to separate the nucleus, 1 × MS buffer added to the abovementioned pellets (containing nuclear components) followed by centrifugation at 700 × *g* for 7 min at 4 °C. The supernatants were removed, and the pellets were lysed in lysis buffer (nucleus).

### Genomic DNA fragmentation

ARPE-19 and RGC-5 cells were treated with 0.5 mM H_2_O_2_ for 6 h and harvested. Genomic DNA was extracted using the QIAamp genomic DNA kit (Qiagen, Hilden, Germany) according to the manufacturer's protocol. DNA extracts were resolved electrophoretically in an 0.8% agarose gel and fragmented DNAs were visualized under ultraviolet light.

### Measurement of the mitochondrial potential

ARPE-19 cells were treated with 0.5 mM H_2_O_2_ with or without 10 *μ*M olaparib for 3 h and then harvested. Mitochondrial membrane depolarization was measured using the Muse MitoPotential kit (Millipore). Briefly, the cells were incubated with Muse MitoPotential dye for 20 min in a 37 °C CO_2_ incubator. Subsequently, changes in the mitochondrial membrane potential were determined with a Muse analyzer (Millipore).

### Measurement of NAD+ and ATP levels

ARPE-19 cells were seeded into 96-well plates (1 × 10^4^ cells/well) and incubated for 12 h. The cells then were treated with 0.5 mM H_2_O_2_ with or without 10 *μ*M olaparib for 4 h. The cellular NAD+ levels were measured using the NAD/NADH-Glo assay kit (Promega Corporation, Madison, WI, USA) according to the manufacturer's instruction. Additionally, the ARPE-19 cells in 96-well plates were treated with 0.5 mM H_2_O_2_ in the presence or absence of 10 *μ*M olaparib for the indicated time points. Cellular ATP levels were measured using the CellTiter-Glo viability assay kit (Promega) according to the manufacturer's instructions.

### Gene silencing with siRNA

Small interfering RNA (siRNA) oligonucleotides were purchased from Bioneer (Daejeon, Korea) with sequences targeting RIPK1 #2 (5′-CACACAGUCUCAGAUUGAU-3′), AIF #1 (5′-GCAAGUUACUUAUCAAGCU-3′) and PARP-1 #3 (5′-GGAGGGUCUGAUGAUAGCA-3′). ARPE-19 cells were transfected with 200 nM of the indicated siRNA or scRNA using Lipofectamine RNAiMAX reagent (Thermo Fisher Scientific) according to the manufacturer's instructions. The effects of siRNA on the indicated protein levels were examined by western blot analysis.

### Animal model

#### Mice

C57BL/6 mice (male, 9 weeks old, weight range: 23–26 g) were purchased from Central Lab Animal (Seoul, Korea). All mice were maintained in the animal facility of Chungnam National University (Daejeon, Korea) and acclimatized to a light schedule of alternating 12-h periods of light and dark with free access to food and water for at least 1 week before the experiment and experimental duration. All animal studies were conducted in accordance with the institutional guidelines for the care and use of laboratory animals. All mice were divided into 4 groups; control (*n*=6), control-olaparib (*n*=6, olaparib 15 mg/kg, i.p), SI-vehicle (*n*=6, SI 30 mg/kg, i.p), SI-olaparib (*n*=6, SI 30 mg/kg, i.p and olaparib 15 mg/kg, i.p). The administrative procedure is shown in Figure 5a.

#### Rabbits

Chinchilla rabbits (male, 3 months, 3 kg) were purchased from Yonam University (Chonan, Korea). All rabbits were maintained in the animal facility of the Catholic University of Korea (Seoul, Korea) and were acclimatized to a light schedule of alternating 12-h periods of light and dark with free access to food and water for at least 1 week before the experiment and experimental duration. All rabbits were divided into 4 groups: control (*n*=3); control-olaparib (*n*=3, olaparib 15 mg/kg, i.p); SI-vehicle (*n*=3, SI 15 mg/kg, i.v); and SI-olaparib (*n*=3, SI 15 mg/kg, i.v and olaparib 15 mg/kg, i.p). The administrative procedure is shown in Figure 6a.

### Protein extraction from the retina

Retinas from enucleated eyes were homogenized in lysis buffer as stated above. Homogenates were centrifuged at 12 000 × *g* for 10 min at 4 °C. Total protein concentrations were determined by the Bradford method. The same volume of proteins was subjected to western blot analysis.

### Hematoxylin and eosin staining

Enucleated eyes were prefixed in 4% paraformaldehyde in PBS at room temperature for 20 min, and the lens was extirpated. Next, the samples were incubated in 4% paraformaldehyde for 12 h and embedded using routine procedures. After embedding, retinal cross sections were prepared with a thickness of 5 *μ*m. The slices were dewaxed, stained with hematoxylin for 5 min, and restained with eosin for 5 min. The samples were observed under an optical microscope (Leica Microsystems, Wetzlar, Germany) and imaged with a slide scanner (Motic Electronic, Xiamen, China).

### Fundoscopic examination

Experimental rabbits were anesthetized using a mixture of ketamine (10 mg/kg, Yuhan, Seoul, Korea) and xylazine (2 mg/kg, Rompun, Bayer AG, Leverkusen, Germany). The rabbits were positioned upright and subjected to pupillary dilation with topical 1% isopto atropine (Alcon Pharmaceuticals, Fribourg, Switzerland). The fundoscopic image of rabbit retina was imaged using a retinal camera (Topcon, Tokyo, Japan).

### Electroretinogram

Rabbits were adapted to the dark for 2 h. The following day, topical Alcaine (Alcon Pharmaceuticals) and 1% isopto atropine were administered to dilate the pupils. The rabbits were then anesthetized according to the same protocol for fundus photography and placed on a heat pad to maintain body temperature during the experiments. The value of the A-wave was measured from 0 to the peak of the initial negative deflection, and the B-wave was measured from the absolute peak of the A-wave to the peak of the positive deflection within 200 ms of the light stimulus. The ERG recording was coordinated using Desktop ERG Viewer software (Ver 3.1, RetVet Corp, Columbia, MO, USA).

### Statistical analyses

At least ⩾3 independent experiments were carried out *in vitro* and *in vivo*. All data are expressed as the mean±S.D. Statistical significance of the experimental and control groups were evaluated using a two-tailed *t*-test. A *P*-value <0.05 was considered significant.

## Figures and Tables

**Table 1:** The ERG parameters of rabbits in the indicated groups

**A-wave parameters**			
	**A-wave (*****μ*****V)**	***P*-value from control-veh**	***P*-value from SI-veh**
Control-vehicle	58.67±9.07	—	—
Control-olaparib	54.67±9.29	0.311	—
SI-vehicle	12.33±1.53	<0.001	—
SI-olaparib	55±2.65	0.269	<0.001

**B-wave parameters**			
	**B-wave (*****μ*****V)**	***P*-value from control-veh**	***P*-value from SI-veh**
Control-vehicle	173.33±37.02	—	—
Control-olaparib	191.67±59.97	0.388	—
SI-vehicle	47.67±12.22	0.002	—
SI-olaparib	209.33±23.71	0.151	< 0.001

Abbreviations: SI, sodium iodate; veh, vehicle
