# Supplementary material for: AIF-independent parthanatos in the pathogenesis of dry age-related macular degeneration
Source: Cell Death Dis. 2017 Jan 5;8(1):e2526–. doi: 10.1038/cddis.2016.437 (PMC5386356; doi:10.1038/cddis.2016.437)

## Supplemental Figure S1

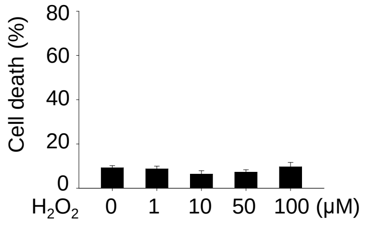

## Supplemental Figure S2

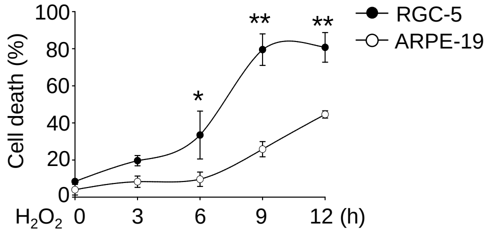

## Supplemental Figure S3

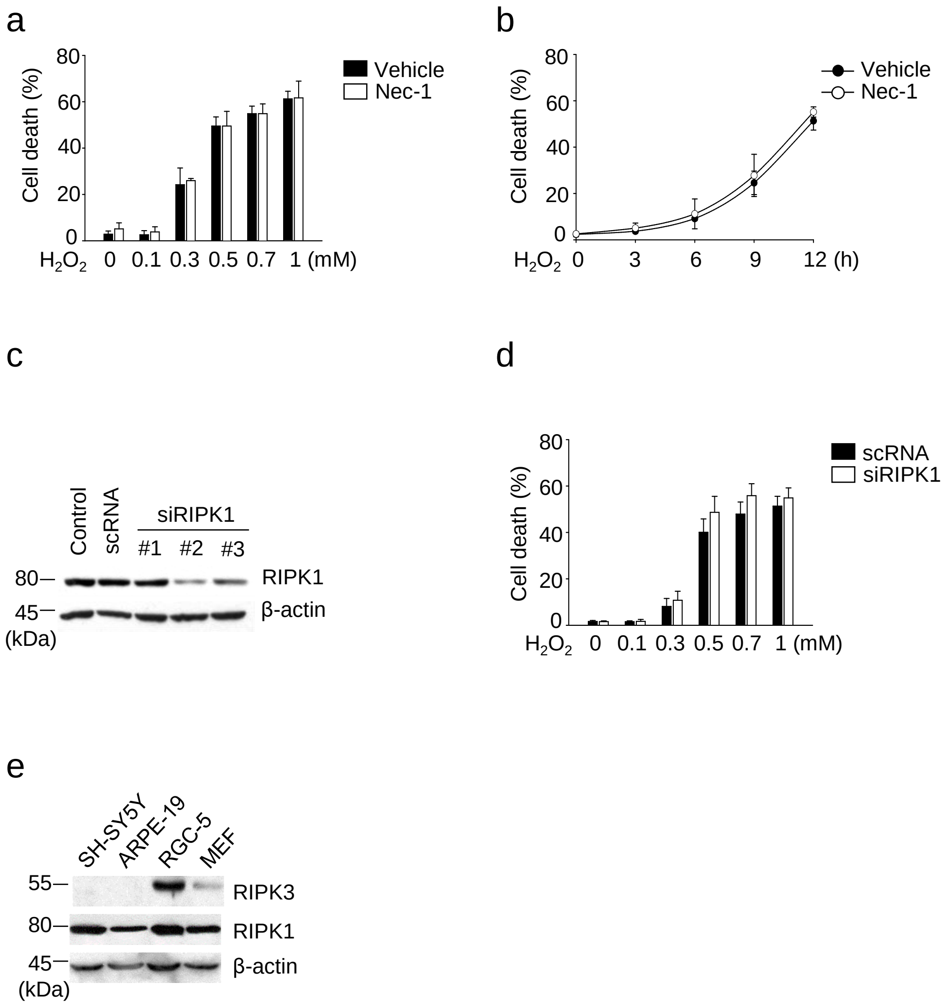

# Supplemental Figure S4

a

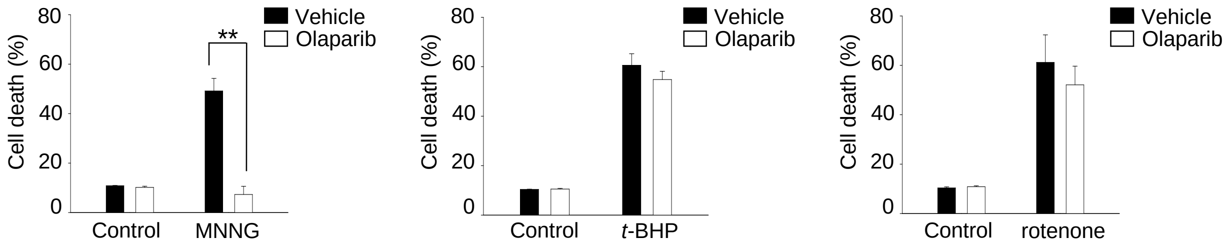


b

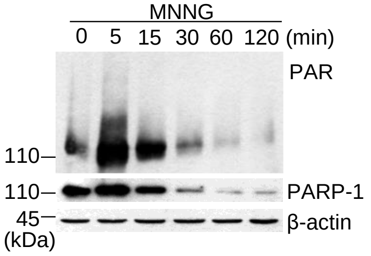

**Supplemental Figure S5**

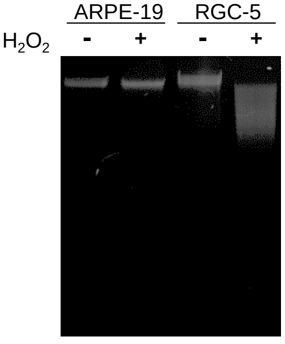

## Supplemental Figure S6

a

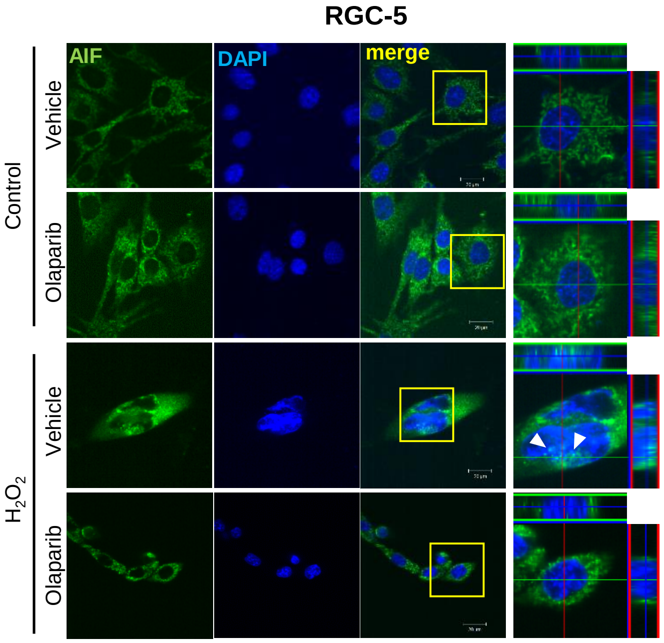


b

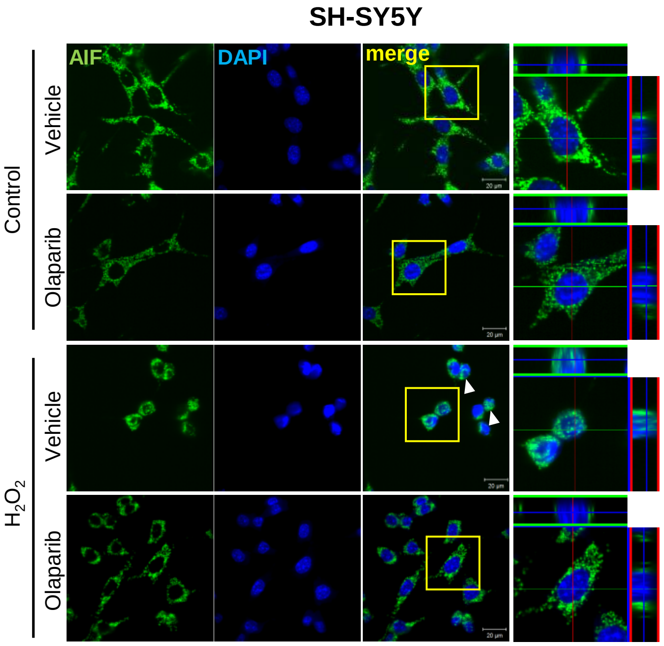

# Supplemental Figure S6

C

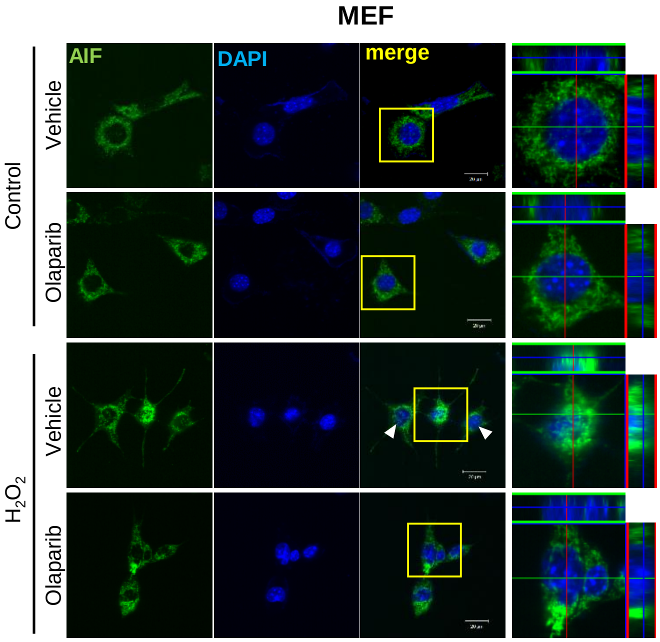

## Supplemental Figure S7

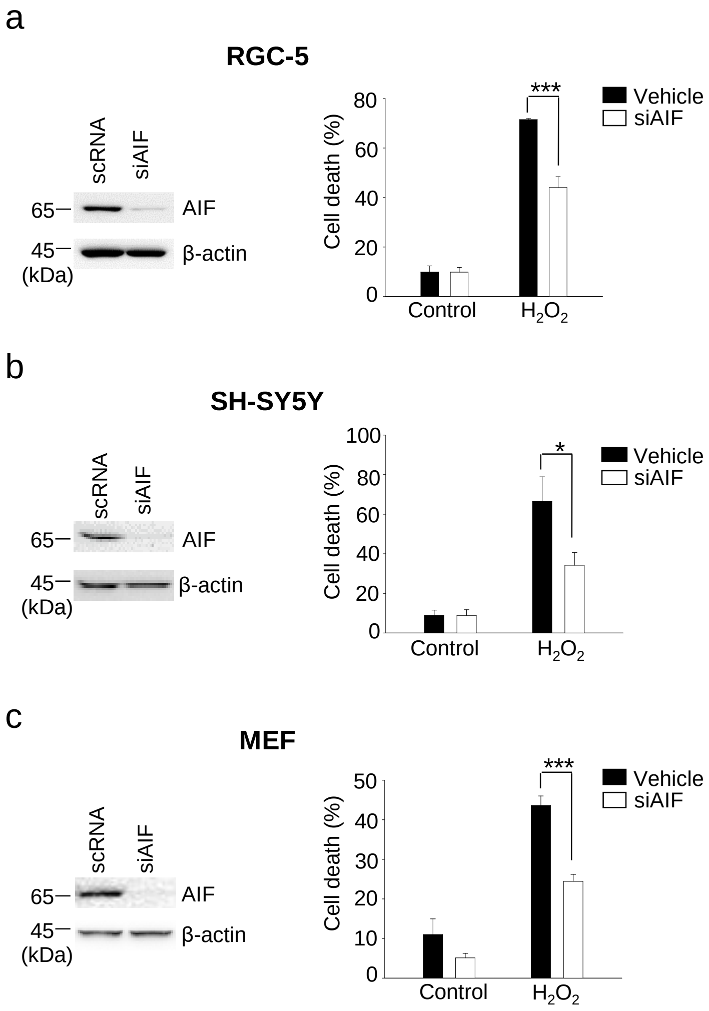

## Supplemental Figure S8

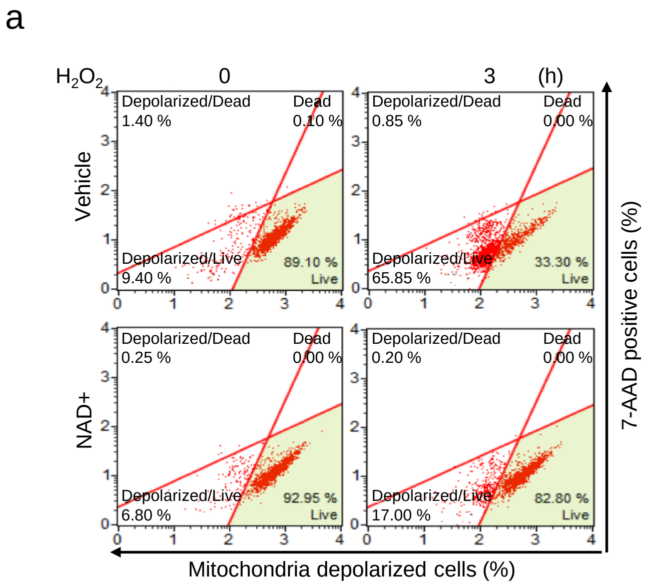


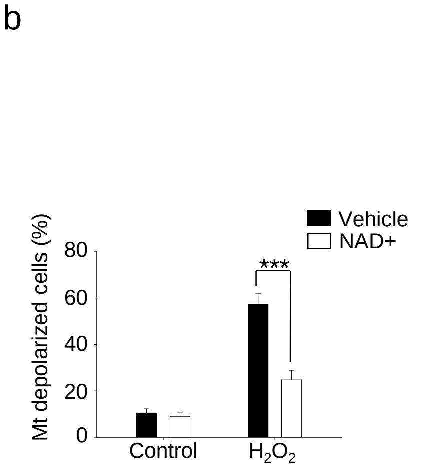

# Supplemental Figure S9

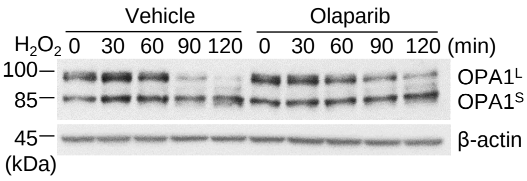

## Supplemental Figure S10

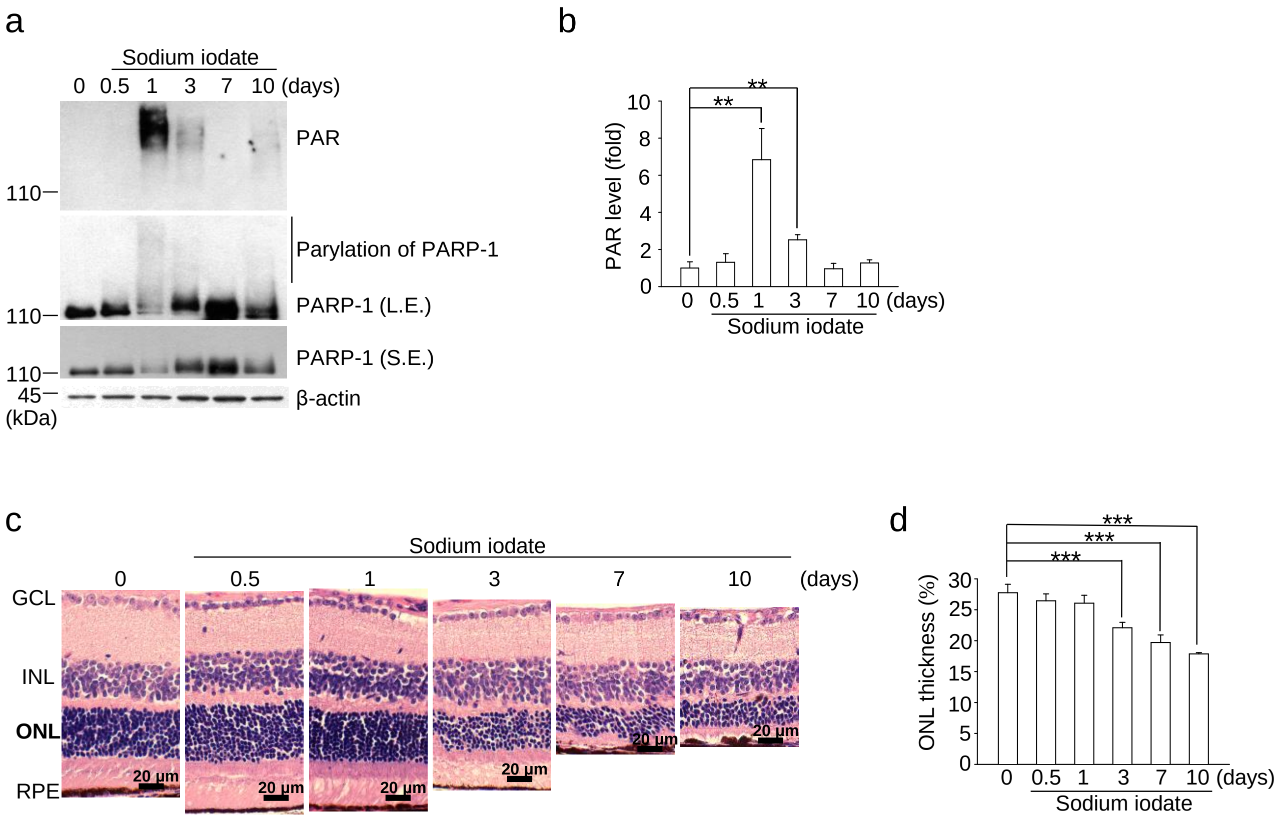

**Supplemental Figure S11**

a

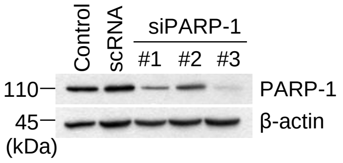


b

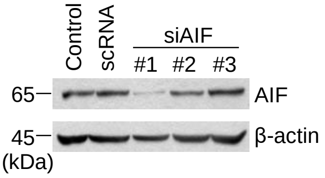

Supplement: Supplementary Figures [file cddis2016437x1.pdf]
