# Supplementary material for: AIF-independent parthanatos in the pathogenesis of dry age-related macular degeneration
Source: Cell Death Dis. 2017 Jan 5;8(1):e2526–. doi: 10.1038/cddis.2016.437 (PMC5386356; doi:10.1038/cddis.2016.437)
Supplement: Supplementary Figure Legends [file cddis2016437x2.docx]

**Supplemental Data**

**Supplemental Figure S1**

Cell death was detected by flow cytometry in ARPE-19 cells treated with low concentrations (1-100 μM) of H_2_O_2_ for 12 h. The values are presented as the mean ± S.D of three independent experiments.

**Supplemental Figure S2**

Cell death was detected by flow cytometry in ARPE-19 and RGC-5 cells treated with 0.5 mM H_2_O_2_ for indicated times. The values are presented as the mean ± S.D of three independent experiments. **P*<0.05, ***P*<0.01.

**Supplemental Figure S3**

(a and b) Cell death analysis in ARPE-19 cells treated with H_2_O_2_ in a dose- (a) and time- (b) dependent manner with or without 20 μM necrostatin-1. (c) RIPK1 expression in ARPE-19 cells transfected with the indicated RIPK1 siRNAs. (d) Cell death analysis in ARPE-19 cells transfected with RIPK1 siRNA (siRIPK1 #2). (e) RIPK3 expression in various cell types*.* The values are presented as the mean ± S.D of three independent experiments.

**Supplemental Figure S4**

(a) Cell death analysis in ARPE-19 cells in response to other oxidative stresses (200 μM MNNG, 150 μM *t*-BHP and 10 μM rotenone for 48 h, 3 h and 72 h, respectively) with or without 10 μM olaparib. (b) PAR polymer expression in ARPE-19 cell following exposure to 100 μM MNNG. The values are presented as the mean ± S.D of three independent experiments. ***P*<0.01.

**Supplemental Figure S5**

ARPE-19 and RGC-5 cells were treated with 0.5 mM H_2_O_2_ for 6 h. Subsequently, genomic DNA was extracted and subjected to gel electrophoresis.

**Supplemental Figure S6**

(a) RGC-5, (b) SH-SY5Y and (c) MEF cells were treated with 0.5 mM H_2_O_2_ for 6 h in the presence or absence of 10 μM olaparib. Immunofluorescence images (including z-stacks) showing the localization of AIF.

**Supplemental Figure S7**

(a) RGC-5, (b) SH-SY5Y and (c) MEF cells were transfected with the AIF siRNA. Indicated left panel showed AIF expression in cells. Cell death was detected by flow cytometry in indicated cells treated with 0.5 mM H_2_O_2_ for 12 h. The values are presented as the mean ± S.D of three independent experiments. **P*<0.05, ****P*<0.001

**Supplemental Figure S8**

(a) ARPE-19 cells were treated with 0.5 mM H_2_O_2_ with or without 1 mM NAD+ for 3 h and the mitochondrial potential was measured using a Muse analyzer. (b) The graph was obtained from a quantitative analysis of depolarized cells. The values are presented as the mean ± S.D of three independent experiments. ****P*<0.001

**Supplemental Figure S9**

The cellular levels of OPA1^S^ and OPA1^L^ in 0.5 mM H_2_O_2_-treated ARPE-19 cells with or without 10 μM olaparib for the indicated time points.

**Supplemental Figure S10**

Time course for SI-injected mice. (a) Immunoblot analysis of PARP-1 and PAR polymers in retina lysates. (b) Quantitative analysis of PAR polymer levels. (c) The retina samples stained with hematoxylin and eosin showing morphological changes over time. (d) Quantitative analysis of the ONL thickness. The values are presented as the mean ± S.D of three independent experiments. ***P*<0.01, ****P*<0.001.

**Supplemental Figure S11**

(a) PARP-1 expression in ARPE-19 cells transfected with the indicated PARP-1 siRNAs. (b) AIF expression in ARPE-19 cells transfected with the indicated AIF siRNAs.
